# The Pneumatic Engine-Improvement

**Published:** 1871-07

**Authors:** 


### THE PNEUMATIC ENGINE-IMPROVEMENT.

We have been using Green's Pneumatic Bur Engine in our practice for about a year and a half, and can speak of its merits with a considerable degree of confidence. Up to about four months ago we used it as it was first presented to the profession, and had become so attached to it that we would sooner have given up any other instrument in use than this. We turned to it as the finishing instrument in almost every operation of filling, and because of its rapid and efficient execution. The work it performs can be better and easier done in one-third the time, than by any other method. It is invaluable also for excavating and forming cavities preparatory to filling, a large proportion of this work we now do with this machine, and here, as in finishing, because it does the work so well and so rapidly.

The instrument with its file carrier—substituting a slip of wood for the file—is equally valuable for cleaning teeth—and here, too, for its rapidity and efficiency. The instrument is valuable in so many respects, that we can not detail them all here. The only objection to the instrument that has at any time been suggested, was its want of power; within the last four months this objection has been wholly removed by a simple change in its gearing, so that now it has all the power that any one could wish for operations upon the teeth; indeed, with the power the instrument now has, care must be exercised, especially by beginners, lest injury be done to the tooth or filling upon which it is brought to bear; an ordinary tooth could be all cut away in a few moments with it.

An improvement has also been made in the cut of the Burs, giving to them more than double their former cutting power. In all the uses to which this instrument is applied, the first thing to be guarded by the beginner, is over execution. No dentist can afford to be without this machine. T.

